# Scale-Up of PTFE-Based Gas Diffusion Electrodes Using an Electrolyte-Integrated Polymer-Coated Current Collector Approach

**DOI:** 10.1021/acsenergylett.4c00114

**Published:** 2024-03-03

**Authors:** Michael Filippi, Tim Möller, Remigiusz Pastusiak, Erhard Magori, Benjamin Paul, Peter Strasser

**Affiliations:** † The Electrochemical Energy, Catalysis, and Materials Science Laboratory, Department of Chemistry, Chemical Engineering Division, Technical University Berlin, 10623 Berlin, Germany; ‡ Siemens Energy (SE) New Energy Business (NEB) Technology & Products (TP) Development (DEV), Siemens Energy Global GmbH & Co. KG, 81739 Munich, Germany

## Abstract

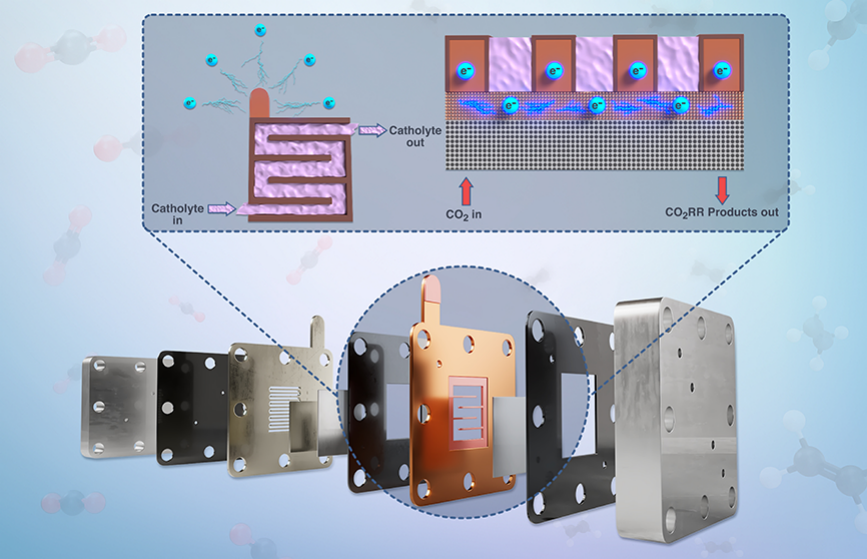

Nonconductive porous polymer substrates, such as PTFE, have been pivotal in the fabrication of stable and high-performing gas diffusion electrodes (GDEs) for the reduction of CO_2_/CO in small scale electrolyzers; however, the scale-up of polymer-based GDEs without performance penalties to technologically more relevant electrode sizes has remained elusive. This work reports on a new current collector concept that enables the scale-up of PTFE-based GDEs from 5 to 100 cm^2^ and beyond. The present approach builds on a multifunctional current collector concept that enables multipoint front-contacting of thin catalyst coatings, which mitigates performance losses even for high resistivity cathodes. Our improved current collector design concomitantly incorporates a flow-field functionality in a monopolar plate configuration, keeping electrolyte gaps small for increased performance. Experiments with 100 cm^2^ cathodes were conducted in a one-gap alkaline AEM and acid CEM system. Our design represents an important step forward in the development of larger-size CO_2_ electrolyzers.

The electrocatalytic CO_2_ reduction reaction (CO_2_RR) is a promising technological platform for the production of value-added chemicals, fuels, and energy storage molecules.^1−3^ To realize this, CO_2_RR electrolyzers need to be developed that fit the industrial needs in terms of high current densities (>200 mA cm^–2^) and stability (≥50.000 h).^4,5^ So far, CO_2_RR electrolyzers that employ gas diffusion electrodes (GDEs) seem to be the most favorable systems for industrial needs, enabling high current densities and faradaic efficiencies (FE) for CO_2_RR products.^6,7^

The biggest issue for implementing electrochemical CO_2_ reduction in industry is the insufficient stability of gas diffusion electrodes caused by multiple degradation mechanisms. Besides the degradation of the catalyst or support involving, among other things, gas bubble-induced wear, the primary failure mechanisms of a GDE have remained flooding and salting.^8−12,21^ Flooding refers to the penetration of the liquid electrolyte into the pores of the GDE, leading to a hindered transport of CO_2_ to the active catalyst sites. This can result in a rapid decline of CO_2_RR selectivity within minutes to several hours, contrasting with the required stability needed for industrial production of ≥50.000 h.

Figure 1 provides an overview of the most common GDE types used in CO_2_RR electrolyzer cells. Carbon-based gas diffusion layers (GDL) form the most frequently used substrates for GDEs (Figure 1A).^13−18^ Key advantages are their conductivity, allowing for scale-up to larger electrode areas. However, a downside of carbon-based cathodes is their tendency for flooding, limiting a stable long-term operation.^19^ To prevent flooding, PTFE-based substrates were introduced for CO_2_RR electrolyzers (Figure 1B).^6,7,20−24^ Their key disadvantage is their lack of conductivity, yet their highly hydrophobic nature prevents excessive flooding. To date, the low conductivity of PTFE-based GDEs has precluded their scalability: Current must be supplied via edge-contacts relying entirely on the in-plane conductivity of the thin catalyst layer, which is why this type of electrode has so far been limited to 1–5 cm^2^ size. Therefore, scale-up of CO_2_RR electrolyzers has only been demonstrated with conductive, e.g., carbon-based, GDEs.^25−29^ A number of different PTFE GDE scale-up concepts have been recently proposed;^6,7,30,31^ however, these approaches were limited to an electrode area of 5 cm^2^ or were plagued by very thick catalyst layers in order to ensure sufficient in-plane conductivity.

**Figure 1:**
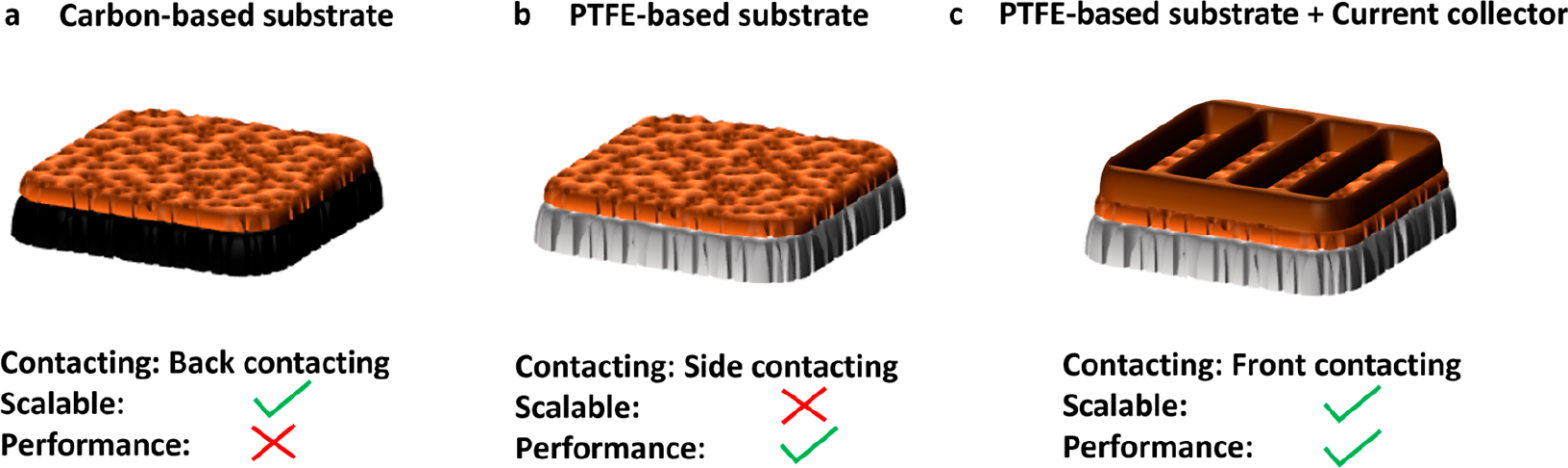
Comparison of different GDE types from the literature using (a) carbon-based substrates, (b) PTFE-based substrates, and (c) PTFE-based substrates with additional current collector.

Here, we introduce a new current collector design that enables reliable scale-up of PTFE based GDEs to 100 cm^2^ and beyond. Our new design is widely applicable to electrolysis or galvanic gas diffusion cells and therefore is not limited to CO_2_ electrolysis. Our current collector concept allows a direct front contact of catalyst layers coated on PTFE-based cathodes, Figure 1C, without penalty in faradaic efficiency in carbon products.

## Grid Current Collector Design and the Effect of Insulating Polymer Coatings

**Figure 2:**
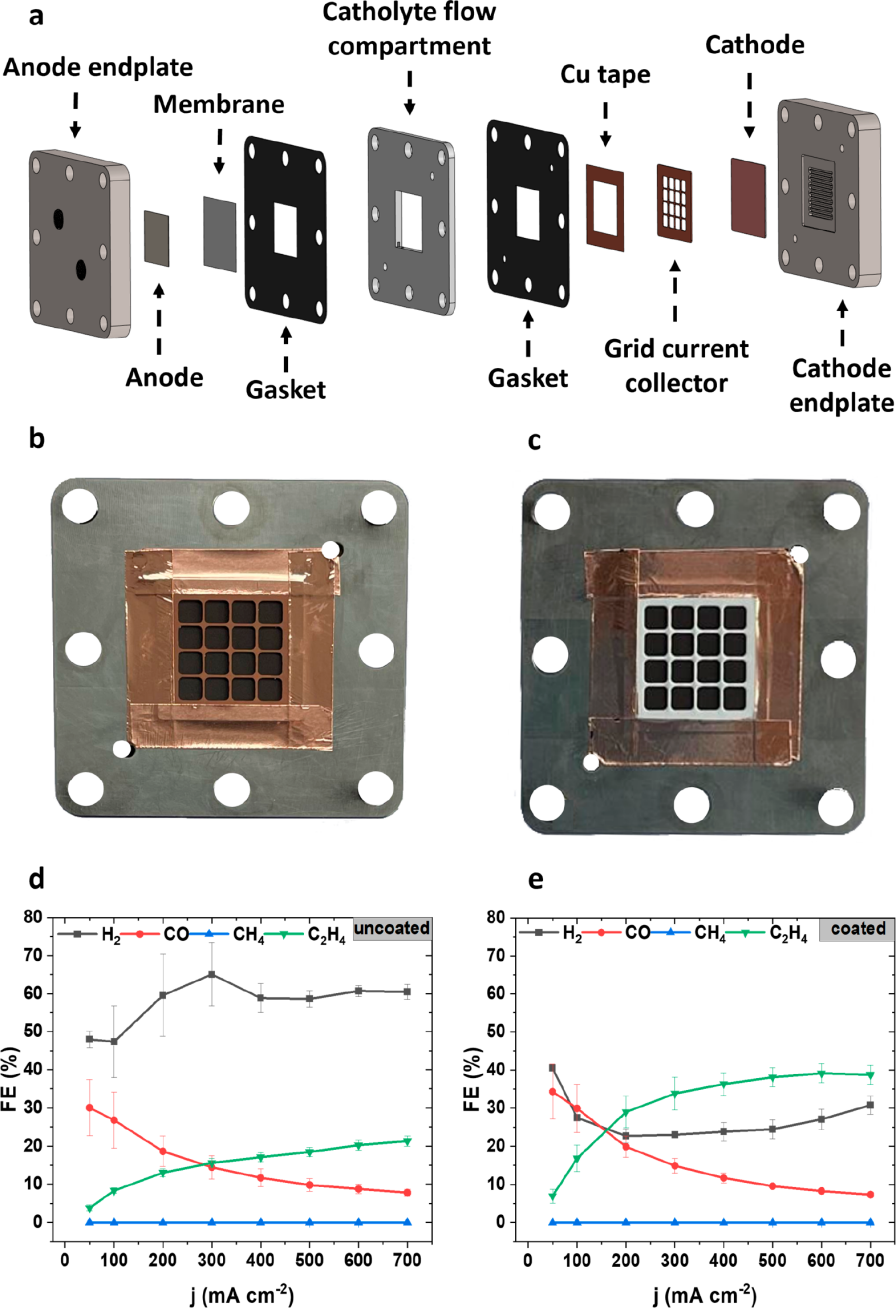
Grid current collector design (5 cm^2^) and the effect of the polymer coating. (a) Exploded view of the cell design with the implemented grid current collector. (b) Photo of the uncoated grid current collector and (c) epoxide-coated current collector installed into the cathode end plate with the catalyst-coated PTFE substrate and the copper tape around the edges for electrical connection. The faradaic efficiencies for the major gas products were plotted on the *y*-axis versus the applied current density on the *x*-axis. The results were obtained on the uncoated grid current collector in (d) and for the epoxide-coated grid current collector in (e).

We propose a new solution for integrating a metallic current collector into electrodes based on insulating substrates. The first generation of our current collector concept was referred to as a “grid” current collector, which essentially is a square-shaped copper plate with 5.22 × 5.22 mm^2^ sized openings. A dedicated electrochemical cell was designed to fit the grid current collector (0.5 mm thickness) in addition to the PTFE-based GDE (0.5 mm thickness), which is shown in more detail in Figure 2a. Details on the electrochemical setup and the cell assembly using this current collector design are provided in Supplementary Figures 1 and 2. A 0.95 mm cavity was CNC-milled into the cathode end plate so that the combination of the grid current collector, which is mounted on the cathode GDE, and the cathode end plate is almost flat, protruding 0.05 mm out of the cavity. This is necessary to guarantee sufficient contact pressure toward the copper catalyst layer for good electrical contact between the current collector and the copper-based catalyst layer. A copper oxide (tenorite) catalyst with 5 wt % Nafion binder and a catalyst loading of 1 mg cm^–2^ was used in this study for preparation of all investigated GDE cathodes. The catalyst was spray-painted on a commercial porous PTFE substrate that is a sintered PTFE membrane with a thickness of 0.5 mm supplied by ElringKlinger. The grid current collector was electrically connected to the cathode end plate via copper tape, as indicated in Figure 2a. A key design feature of the copper-based grid current collector was an epoxide coating, Figure 2c. Additional views of the coated and uncoated grid current collector without the surrounding end plate and copper tape can be seen in Supplementary Figure 3. The epoxide coating was applied via an electrostatic powder coating and a subsequent sintering approach. We emphasize that the epoxide-coated current collector was coated only in the places where the current collector is in contact with the electrolyte, which means that the surfaces in contact with the catalyst layer and copper tape were free of the epoxide coating.

Figure 2d and Figure 2e show the detected gas product distribution as a function of applied current density for CO_2_ electrolyzer cells incorporating an uncoated and epoxide-coated current collector, respectively. For both experiments, 1.0 M KHCO_3_ was used as the catholyte and anolyte. Ni foam was used as the anode in direct contact with the AEM membrane (Selemion, AMV). In the case of the uncoated current collector, a high amount of hydrogen (>50% FE) is produced throughout the full current density range with a maximum of approximately 20% FE of ethylene at 700 mA cm^–2^. On the other hand, the coated current collector produced almost twice as much ethylene at 700 mA cm^–2^ and less than half the amount of hydrogen in the 200–700 mA cm^–2^ current density range. The insulation of the metallic current collector surfaces using the epoxide polymer was evidently essential to suppressing the hydrogen evolution reaction (HER) and increasing the ethylene faradaic efficiency at the same time. The origin of the enhanced undesired hydrogen evolution on the uncoated current collector is the portion of the current collector surface fully immersed in the electrolyte, where only a small concentration of CO_2_ is available, which limits the production of CO_2_RR products. In other words, an uncoated metallic current collector partially acts as an extended unselective catalyst surface as well as a current collector. Once the current collector surface gets passivated with an electrically insulating material, such as epoxide polymer, the current collector acts exclusively as a current distributor.

The key advantage of our current collector design is that it is possible to use very thin catalyst layers without sacrificing the electrode conductivity and CO_2_RR performance. By contrast, a recent carbon overlayer approach by Dinh et al. required very thick carbon overlayers, which could lead to severe selectivity issues, comparable to our uncoated current collector system (Figure 2d).^6,7^ Additionally the potential response after the application of a current starting from the open circuit potential is much better in the case of our current collector approach in comparison to the typical side contacting approach, where a markable potential overshoot and slow reduction of the CuO phase can be seen (Supplementary Figure 4). The enhanced potential response due to compartmentalization of the electrode into smaller fragments also enables the scale-up toward larger electrode sizes without sacrificing the performance of the electrode due to issues in conductivity.

## Monopolar Plate Current Collector Design for High-Performance CO_2_RR Electrolyzers

**Figure 3:**
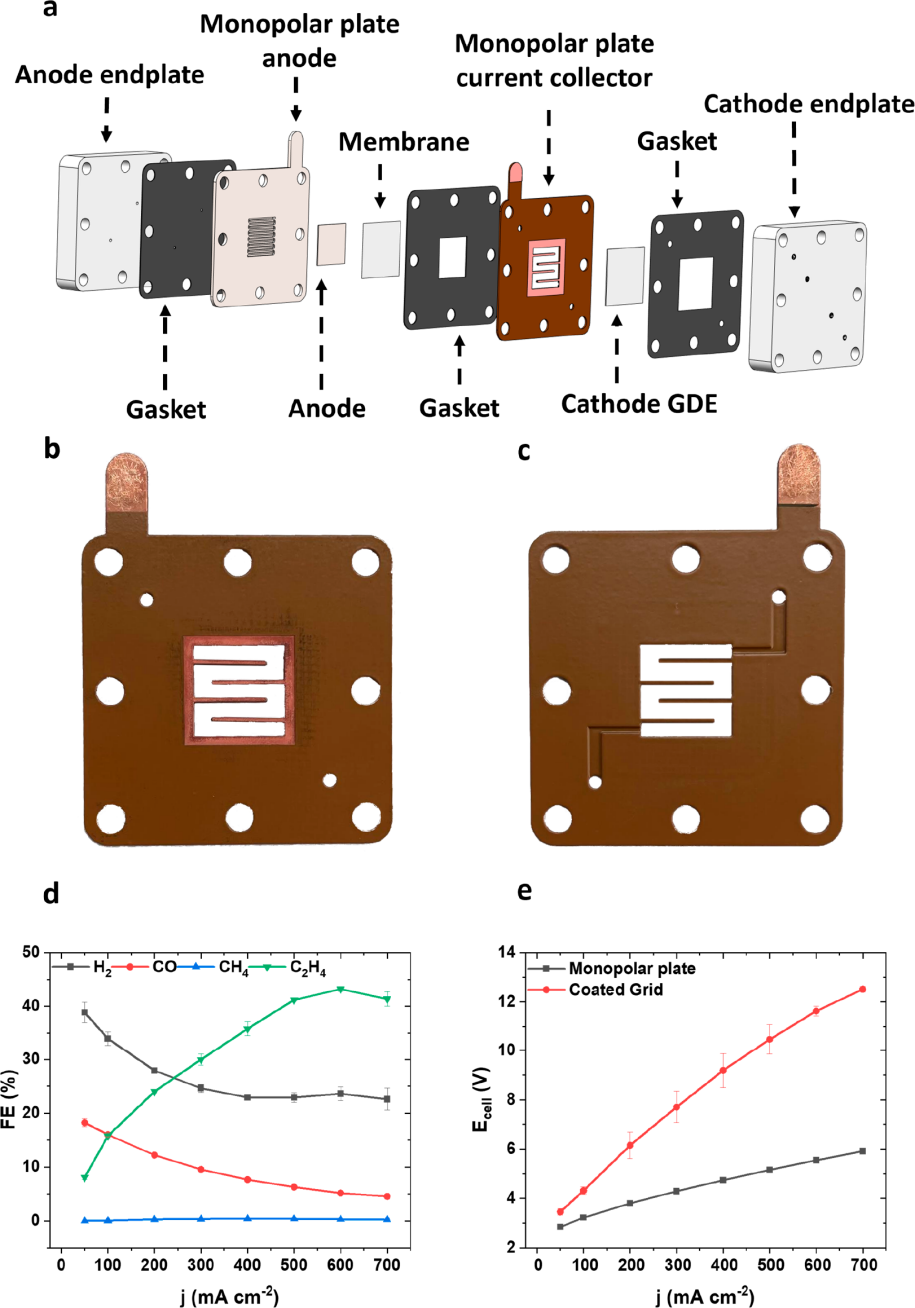
Monopolar plate current collector design (5 cm^2^). (a) Exploded view of the cell with the implemented monopolar plate current collector. (b) Photo of the PEEK-coated monopolar plate current collector from the back side (facing the catalyst layer) and (c) from the front side (facing the membrane). (d) Faradaic efficiencies for the major gas products plotted on the *y*-axis versus the applied current density on the *x*-axis. (e) Polarization curves attained with the grid current collector (“coated grid”) cell design in red and the monopolar plate design (“monopolar plate”) in black.

While the monofunctional grid current collector design in Figure 2a enables geometric scale-up, its presence in the cell assembly increases the anode-to-cathode distance and, therefore, cell resistance, limiting cell performance. Therefore, in an advanced current collector design, we integrated the current collector functionality into the electrolyte flow compartment, resulting in the cell design of Figure 3.

Figure 3a shows single cell components including the new monopolar plate current collector design, and images of the cell assembly are provided in Supplementary Figure 5. The monopolar plate current collector is a serpentine liquid flow compartment made from copper with an additional polymer coating. Here, a PEEK coating (brown color) was used instead of epoxide due to PEEK’s much higher mechanical and chemical resistance. The monopolar plate current collector was manufactured by CNC milling a 1 mm thick copper plate with consecutive PEEK powder coating and sintering. The PEEK polymer coating had a total thickness of 100 μm, which resulted in a total current collector thickness of 1.2 mm. After the PEEK polymer coating, an engraving laser machine was used to remove the PEEK polymer in the places where an electrical contact had to be established. For better clarity, images of the monopolar plate current collector are provided in Figure 3b,c. Additional images showing the monopolar plate current collector from CNC machining to the lasering steps are provided in Supplementary Figure 6. A 27 × 27 mm^2^ sized square was removed on the backside of the monopolar plate (Figure 3b), where the catalyst layer is in contact with the current collector.

A noninsulated electrical connection was added on the top of the current collector, to establish the connection to the external power supply. Figure 3c shows the front side of the monopolar plate current collector, where the coated flow channel ribs and electrolyte inlet and outlet channels are visible. The front side faces the membrane, as can be seen in Figure 3a. The monopolar plate design is superior to the grid design because, besides the reduction of the catholyte gap, the flow channel ribs in the center provide additional mechanical support for the membrane, anode, and cathode. Also, the resulting contact pressure between the copper catalyst and the current collector is more homogeneous in the monopolar plate design compared to the grid current collector design, due to the direct contact and compression of the anode-side components (Ni foam, membrane) and the cathode-side components (monopolar plate, GDE). This in turn would lead to a more homogeneous current distribution in the catalyst layer.

The experimental validation of the superior performance of our new monopolar plate current collector design is shown in Figure 3d,e. Identical experimental conditions as for the grid current collector design tests were employed. The faradaic efficiencies for major gas products compared very well between the two designs. Additionally liquid product production was analyzed in the monopolar design, which is shown in Supplementary Figure 7. However, the key advantage of the monopolar plate design is the significantly reduced applied cell voltage (and hence power requirements) over the current density range, Figure 3e. At 700 mA cm^–2^, the cell voltage more than halved for the monopolar plate design. This was largely due to a reduced anode-to-cathode distance from 4 to 1.2 mm for the grid and integrated monopolar plate current collector, respectively. The reduced gap size thereby translated into a thinner liquid electrolyte layer gap, accounting for improved cell performance.

## Scale-Up of PTFE-Based GDEs from 5 cm^2^ to 100 cm^2^

Using our PEEK-coated monopolar plate current collector design, we successfully scaled PTFE-based GDEs for CO_2_ electrolyzers from 5 cm^2^ and to 100 cm^2^ active geometric area. To the best of our knowledge, this is the first report on a PTFE-based GDE of that size that delivers cell performances matching or exceeding those of 5 cm^2^ cells.

**Figure 4:**
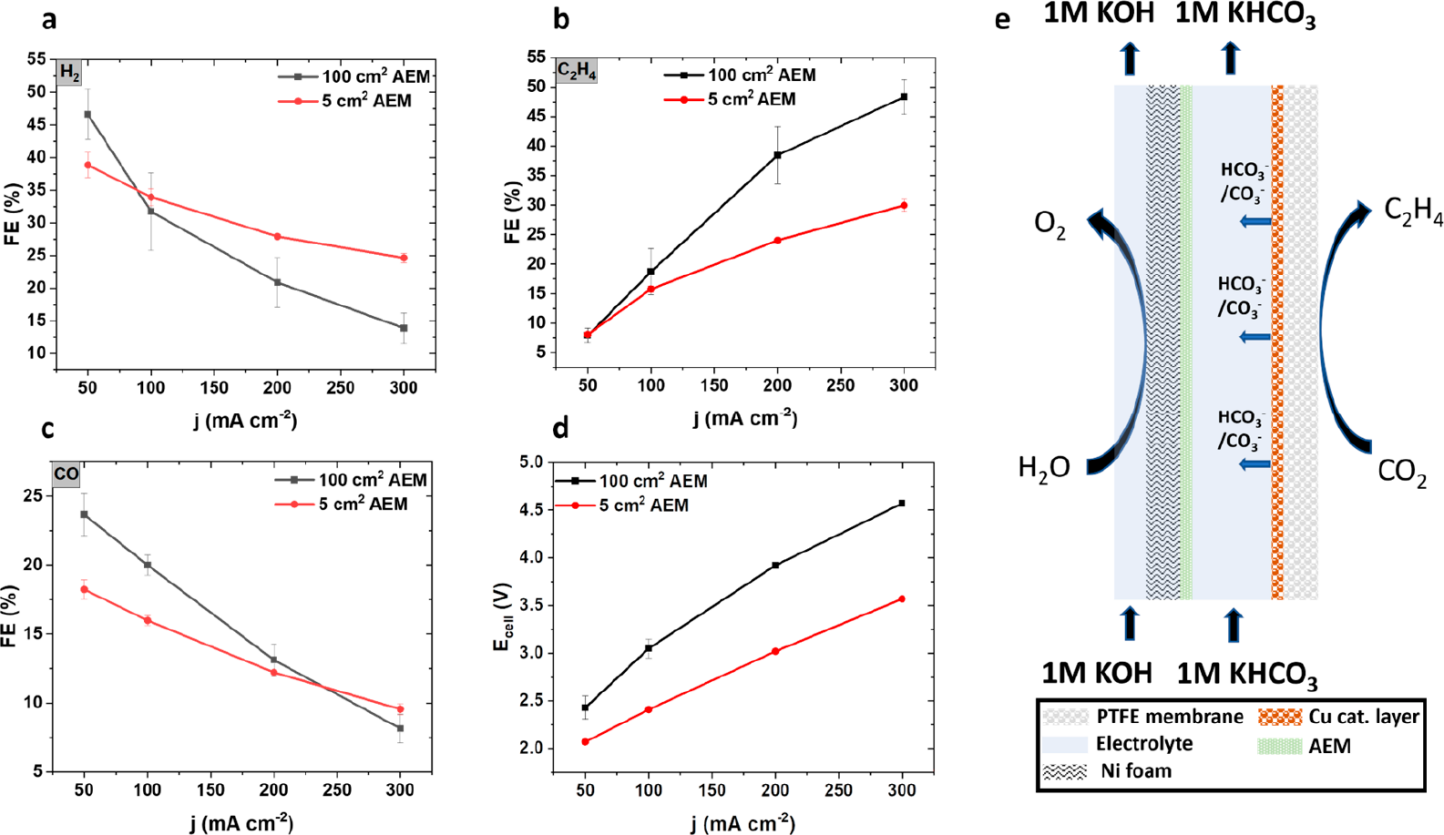
100 cm^2^ scale-up experiments in an AEM cell setup. The results obtained in the 100 cm^2^ cell (black lines) are compared against the results obtained in the 5 cm^2^ cell (red lines). In both cells, the same electrode materials were used. Faradaic efficiency (FE) is plotted on the *y*-axis for (a) hydrogen, (b) ethylene, and (c) carbon monoxide against current density (*j*) on the *x*-axis. (d) Polarization curves are demonstrated, plotting the cell voltage (*E*_cell_) on the *y*-axis against current density (*j*) on the *x*-axis. (e) A schematic that shows ion transport and product generation on the anode and cathode.

Details on the 100 cm^2^ cell setup, cells and the coated GDEs are shown in Supplementary Figures 8–10 and Figure 4e. Clearly, identical cell components and specifications, such as anode-to-cathode distance, were used for a valid comparison to 5 cm^2^ data in Figure 3. For the manufacturing of the current collector, stainless steel was used as a base material, which after CNC milling was coated with a 10 μm thick layer of copper via galvanic deposition. After that, the previously described PEEK powder coating and sintering procedure was applied with the final selective removal of PEEK coating to establish the electrical connection to the catalyst layer and the power source. Additionally, the flow geometry was changed from serpentine to parallel to reduce the fluid velocity inside the catholyte gap and establish a better comparison to the 5 cm^2^ sized system. Finally, 1 M KOH was chosen as anolyte in both 5 cm^2^ and 100 cm^2^ to prevent anode corrosion (further information on the corrosion issue in Supplemental Discussion 1 and Supplementary Figure 11).

The experimental performance of our 100 cm^2^ PTFE-GDE CO_2_-C_2+_ electrolyzer is compared to the 5 cm^2^ analog in Figure 4a–c. Despite a moderate voltage penalty, the faradaic ethylene efficiency increased significantly to about 50% up to 300 mA cm^–2^. This is a previously unachieved value for 100 cm^2^ PTFE-based GDE cells.

Closer inspection showed that less hydrogen was produced in the 100 cm^2^ cell, which we attribute to higher local pH build-up. This observation agrees with the mass transport model from Blake et al., who predicted similar effects in scaled-up systems.^32^ Accordingly, the 100 cm^2^ cell also outperformed the 5 cm^2^ cell in ethylene production, as seen in Figure 4b. At 300 mA cm^–2^, the ethylene FE is almost twice as large in the 100 cm^2^ cell compared to the 5 cm^2^ cell, which again can be explained by higher alkalinity in the catholyte gap. The production of CO, shown in Figure 4c, is enhanced compared to the smaller cell until 300 mA cm^–2^, at which point the production of CO in both cells converges to roughly the same value.

The difference in cell voltage was approximately 360 mV at 50 mA cm^–2^ and rose to 1 V at 300 mA cm^–2^. We attribute this discrepancy to the suboptimal anolyte flow design, evident in the experiments where 1 M KHCO_3_ was used as anolyte in the 100 cm^2^ cell system.

## Performance of Monopolar Plate Current Collector Design in Acidic Proton Exchange Membrane Electrolyzer Cells

**Figure 5:**
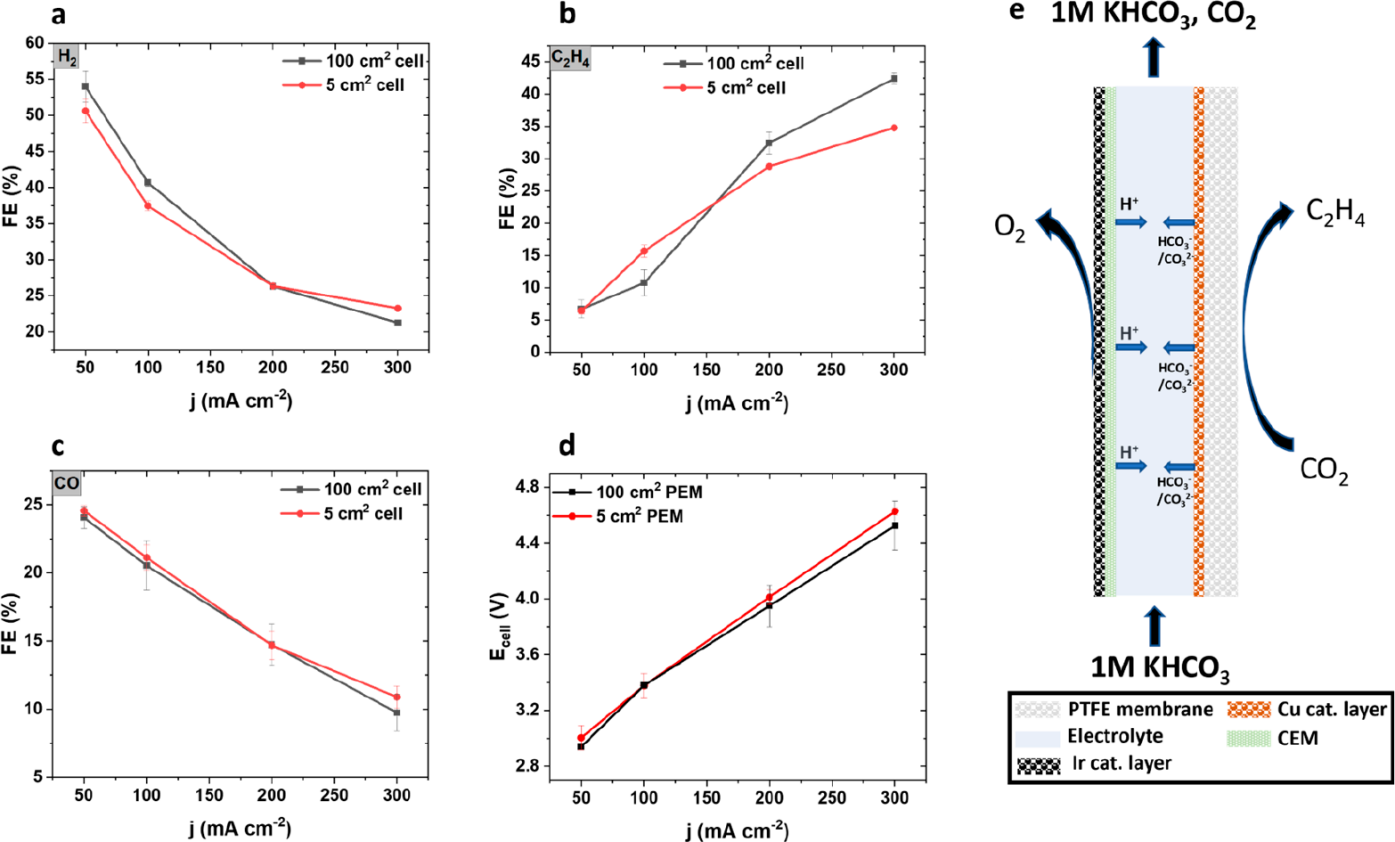
100 cm^2^ scale-up experiments with half-MEA setup. 5 cm^2^ cell (red) results and 100 cm^2^ cell (black) results are compared. Both cells used the same electrode materials. Faradaic efficiencies are plotted on the *y*-axis for (a) hydrogen, (b) ethylene, and (c) carbon monoxide. The current density *j* is plotted on the *x*-axis. (d) Polarization curves are demonstrated, plotting the cell voltage (*E*_cell_) on the *y*-axis against current density (*j*) on the *x*-axis. (e) The schematic shows ion transport and product generation on the anode and cathode.

To further verify the scalability of our coated current collector approach for use with PTFE-based GDEs, we modified the cell design, as shown in the schematic in Figure 5e. To avoid the problem of inhomogeneous anolyte fluid flow and the resulting cell voltage increase, a system was chosen where the anode could be operated in a dry state. An Ir-coated cation exchange membrane (CEM), referred to as a “half-MEA”, was used for these validation experiments.^33^ The water necessary for the anodic oxygen evolution reaction was delivered via back-diffusion of water molecules from the 1 M KHCO_3_ catholyte to the Ir catalyst layer. Now, unlike in the AEM-based CO_2_ electrolyzer system above, there is a CO_2_ gas bubble formation in the catholyte gap due to the protonation of bicarbonate ions by protons crossing through the CEM.

In Figure 5a–c, the FE values of the major gas products for both 5 and 100 cm^2^ half-MEA PEM CO_2_ electrolyzer cells are reported. We provided additional data on current hold experiments for 160 min at 300 mA cm^–2^ for both CEM and AEM systems in the 100 cm^2^ cell format, indicating a stable and reproducible performance in the investigated time frame (Supplementary Figure 12). Unlike in the AEM setup, the H_2_ FE (Figure 5a) and CO FE (Figure 5c) are almost identical in either cell size, suggesting a similar chemical reaction environment at the catalyst surface. The migration of protons to and the associated evolution of CO_2_ bubbles inside the catholyte gap induced additional fluid convection and presumably reduces the local alkalinity build-up along the catholyte gap channel, resulting in a comparable amount of H_2_ and CO selectivity. The ethylene FE in Figure 5b turned out superior between 200–300 mA cm^–2^ for the 100 cm^2^ cell, yet lower in the 50–100 mA cm^–2^ range. Now, again unlike in the AEM electrolyzer, the cell voltages for either cell size were almost identical, supporting our earlier hypothesis that the inhomogeneous anolyte flow in the AEM CO_2_ electrolyzer led to the higher cell voltages. Again, our half-MEA electrolyzer results in Figure 5 impressively demonstrate the robust scalability of our polymer-coated current collector design for PTFE-based GDE cells.

We have reported a novel monopolar plate current collector design with an integrated liquid electrolyte compartment that enables a manifold geometric scale-up of the active electrode area of nonconductive PTFE-based GDEs in electrolyzers. We consider the successful scaling of nonconductive GDEs to technologically more relevant geometric surface areas as an important step forward for the future development of electrochemical cells for production of e-fuels or e-chemicals.

More specifically, the new current collector was demonstrated in a PTFE-GDE of a CO_2_-to-ethylene electrolyzer cell, where it allowed the previously elusive successful scale-up from a 5 cm^2^ to 100 cm^2^ electrode area at comparable or even improved ethylene performance. The partial polymer coating of the current collector surfaces in contact with bulk electrolyte proved crucial to maintaining the CO_2_ selectivity.

We developed our polymer-coated current collector approach starting from an initial grid current collector design and evolved this into our optimized electrolyte-integrated monopolar PEEK-coated current collector design, thereby combining the functionality of the current collector with the function of an electrolyte flow field. Finally, we implemented the integrated current collector design into a modified 100 cm^2^ Electrocell and proved the scalability of our approach for electrochemical CO_2_ reduction cells evidencing, for the first time, high ethylene yields in PTFE electrodes of that size.

Our approach is expected to enable scale-up of other electrolyzer cells, either gas-consuming or gas-producing ones, utilizing a liquid electrolyte channel. We expect our design to be superior in situations where hydrophobic, nonconductive porous substrates constitute essential mass transport enabling cell components, such as in electrochemical cells for the electrochemical activation of N_2_ or the electrochemical co-reduction of CO_2_ and NO_*x*_ compounds to C–N compounds and others.

## Supporting Information Available

GDE and cell component manufacture, product detection, experimental descriptions (electrochemical protocols, components of the setup), images of the setups, and discussion on anode (Ni foam) stability in 1 M KHCO_3_ (PDF)
