# Supplementary material for: Scale-Up of PTFE-Based Gas Diffusion Electrodes Using an Electrolyte-Integrated Polymer-Coated Current Collector Approach
Source: ACS Energy Lett. 2024 Mar 3;9(4):1361–8. doi: 10.1021/acsenergylett.4c00114 (PMC11019647; doi:10.1021/acsenergylett.4c00114)
Supplement: Supplementary file 1 — nz4c00114_si_001.pdf [file nz4c00114_si_001.pdf]

Supplemental Information

Scale-Up of PTFE-Based Gas Diffusion Electrodes Using an Electrolyte-Integrated Polymer-Coated Current Collector Approach

Michael Filippi[1], Tim Möller[1], Remigiusz Pastusiak[2], Erhard Magori[2], Benjamin Paul[1] and Peter Strasser[1]*

[1]The Electrochemical Energy, Catalysis, and Materials Science Laboratory, Department of Chemistry, Chemical Engineering Division, Technical University Berlin, 10623 Berlin, Germany

[2]Siemens Energy (SE) New Energy Business (NEB) Technology & Products (TP) Development (DEV), Siemens Energy Global GmbH & Co. KG, 81739 Munich, Germany

[*]Corresponding author:

Peter Strasser, pstrasser@tu-berlin.de

Methods

Electrochemical cell and electrode preparation. A copper oxide (tenorite) catalyst from Johnson Matthey was used on the cathode for all systems discussed here. This electrode configuration was also used in our previous work.[21] The PTFE substrate used for all CO_2RR measurements was a sintered PTFE membrane (ElringKlinger, 0.5 mm thickness). For the manufacturing of the catalyst coated PTFE membrane, a spray coating approach was chosen, and typical ink composition for example:

- 25 ml of a water/isopropanol (80:20) mixture
- 625 mg CuO powder
- 32.875 mg Nafion

During the spray coating process, the PTFE substrate was heated to 80°C via a hotplate. The catalyst loading of 1.0 mg cm^{-2} was verified by weighing the PTFE membrane before and after spray coating. Electrodes for the 5 cm^2 current collector cell versions were sprayed first to 10 cm^2 geometric area and were later cut to 27 x 27 mm^2 to use the edges of the layer for better current collection and leak tightness. The anode material in all AEM-based setups was a Ni foam (Fraunhofer IFAM, 0.45 mm thickness) and an anion exchange membrane (Selemion, AMV).

Details on the grid current collector system

The design of the grid current collector and all other custom-made current collector designs and cell parts were made with a CAD program from Solidworks (Solidworks 2021). The copper

grid current collectors were manufactured via laser cutting of 0.5 mm thick Cu plates by the company Metall Ehrnsberger. A custom cathode endplate with a 0.95 mm cavity was also manufactured via CNC milling to fit in the PTFE-based GDE and the current collector. An epoxide coating with a thickness of 100 µm was applied via powder coating and sintering by the company Stüwe. The parts of the current collector that had to be electrically connected to the catalyst layer and to the endplate were covered with tape during the coating process. For additional images of the grid current collector cell system see Supplementary Figure 2-3.

Details on the polymer-coated current collector system

The monopolar plate current collector was manufactured by CNC milling of a 1 mm thick copper plate by the company Protolabs. Contrary to the grid current collector, a PEEK polymer coating was applied due to the much higher durability of the coating. A 100 µm thick PEEK coating was applied via PEEK powder coating and sintering by the company acs Coating Systems. Finally, the surfaces needed for the electrical connection to the catalyst layer and the potentiostat were removed via lasering with an engraving laser machine (BN laser, JEWEL liner). The PEEK endplates and anode monopolar plate were also manufactured by CNC milling of PEEK and titanium plates. For the half-MEA measurements, a custom made titanium expanded metal was used as porous transport layer (PTL) in combination with an iridium-coated CEM from Siemens Energy.

Details on the 100 cm^2 cell system

A modified MP Electrocell was used, and a monopolar plate current collector design was implemented for PTFE-based GDEs. In contrast to the 5 cm^2 version of the monopolar plate current collector, a parallel arrangement of the current collector rods was chosen to reduce the fluid velocity of the catholyte (Supplementary Figure 9). The current collector was manufactured via CNC milling of stainless steel by the company Protolabs. In this case,

stainless steel was chosen instead of copper as the base material due to the higher stiffness of the current collector rods. After that, the stainless steel current collector was coated with copper (approximately 15 µm) via galvanic deposition by the company Rudolf Clauss. Finally, the current collector was PEEK powder coated and sintered, and the necessary surfaces for electrical contacts were removed with an engraving laser machine.

Electrochemical setup – 5 cm^2.

An experimental setup, as shown in Supplementary Figure 1, was utilized to test the grid and monopolar plate current collector design. For further details on our setup we refer to our previous publication.[21] A constant CO_2 flow of 50 ml min^{-1} was used for the experiments shown in Figure 2,3. For the comparison between 5 and 100 cm^2 electrolyzers the CO_2 flow was reduced to 25 ml min^{-1} for a better comparison. Mass flow controllers (Bronkhorst) were used to adjust the volumetric flows precisely. A defined flow of nitrogen was introduced before the product gas entered the GC as internal standard, for accurate determination of the faradaic efficiencies of the gas products. Additionally, a separate CO_2 flow described by our previous publication was used to purge the catholyte and detect gas products that cross over into the catholyte.[21] For anolyte and catholyte, 1 M $KHCO_3$ (VWR , ≥99.5%) electrolyte was used in all experiments except for the scale-up comparison experiments where 1 M KOH (VWR, 85 – 100.5%) was used in the AEM configurations as anolyte. The anolyte and catholyte were constantly circulated via separate membrane pumps (KNF, SIMDOS 10) at a flow rate of 50 ml min^{-1}. An exception was made for the monopolar plate current collector design experiments, where a flow of 20 ml min^{-1} was used for the catholyte flow due to the much smaller catholyte gap size compared to the grid current collector design. Only the catholyte was circulated for the half-MEA measurements with the monopolar plate current collector system, and the anode compartment was dry. Back pressure regulators separately controlled the gas and catholyte pressures (Bronkhorst). In all experiments, a 100 mbar higher pressure on the catholyte side

compared to the gas side was utilized. The absolute pressure on the liquid side was 1.2 bar, and 1.1 bar on the gas side. All experiments were performed at room temperature. A Potentiostat (Biologic, SP-150) plus a current booster (Biologic, VMP-3) were used for the electrochemical measurements.

Electrochemical setup – 100 cm^2

For the scale-up experiments a modified MP Electrocell was used to implement PTFE-based electrodes with 100 cm^2 geometric surface area (image of the electrode in Supplementary Figure 10). The setup for the scale-up experiments is shown in Supplementary Figure 8. A constant CO_2 flow of 500 ml min^{-1} was used for the experiments and a separate nitrogen flow as internal standard as described for the 5 cm^2 setup. The CO_2 exit flow was also used to purge the catholyte reservoir for accurate total gas product detection. Mass flow controllers (Sensirion) were used to adjust the flow of CO_2 and N_2. Water columns were used to control the pressure as indicated in the 5 cm^2 setup instead of electronic back pressure regulators. Two separate centrifugal pumps (Flojet) circulated anolyte and catholyte at a flow rate of 400 ml min^{-1}. Electrolytes were the same as in the 5 cm^2 experiments. The catholyte temperature was controlled to room temperature for a good comparison to the 5 cm^2 experiments via a thermostat (Julabo). A power supply (TDK-Lambda, Z10-72) was used for the galvanostatic measurements and the cell potentials were additionally verified with a multimeter (Fluke).

Electrochemical measurement protocol.

Galvanostatic measurement protocols were established for all experiments and the resulting cell potential was measured. For the 5 cm^2 systems (grid current collector and monopolar plate design), measurements were performed with the following current density steps:

50, 100, 200, 300, 400, 500, 600 and 700 mA cm^{-2}.

Each current density step was held for 20 minutes. For the 100 cm^2 cell system the measurements were stopped at 300 mA cm^{-2}. Also, the individual current density steps were held for 1 h instead of 20 min.

Quantification of products

Gas products were quantified with a gas chromatograph from Shimadzu (GC 2014 series) for the 5 cm^2 scale experiments and a gas chromatograph from Agilent (7890A) for the 100 cm^2 scale experiments. Both gas chromatographs were equipped with thermal conductivity detectors (TCD) and flame ionization detectors (FID). The TCD was used for the detection of N_2 and H_2. The FID was used for the detection of CH_4, C_2H_4 and CO. As mentioned above, a specific flow of N_2 was used as an internal standard for accurate determination of the flow entering into the GC.

Liquid products (ethanol, propanol and allyl alcohol) were quantified with a liquid injection gas chromatography system equipped with an FID (Shimadzu, GC 20210 series). Carbonic acids like formic acid and acetic acid were analyzed by high-pressure liquid chromatography (Agilent, 1200 series).

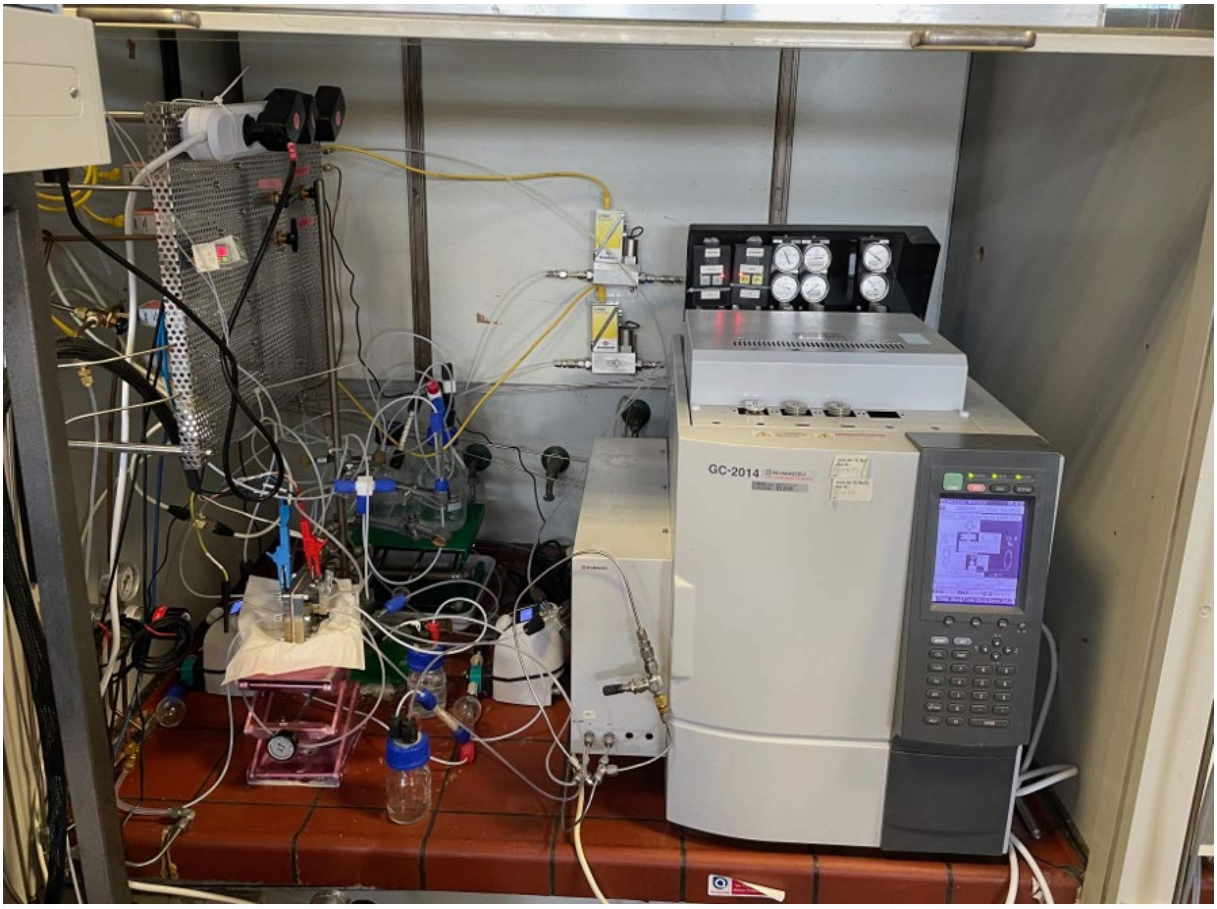

Supplementary Figure 1. Experimental setup for the electrochemical CO_2 valorization in single 5 cm^2 flow electrolyzers operating in alkaline or acidic environments, using nanostructured unsupported CuOx powder catalyst layers deployed on PTFE Gas Diffusion Layers, implementing either *the grid current collector design* or *the monopolar plate current collector design with integrated liquid flow chamber*.

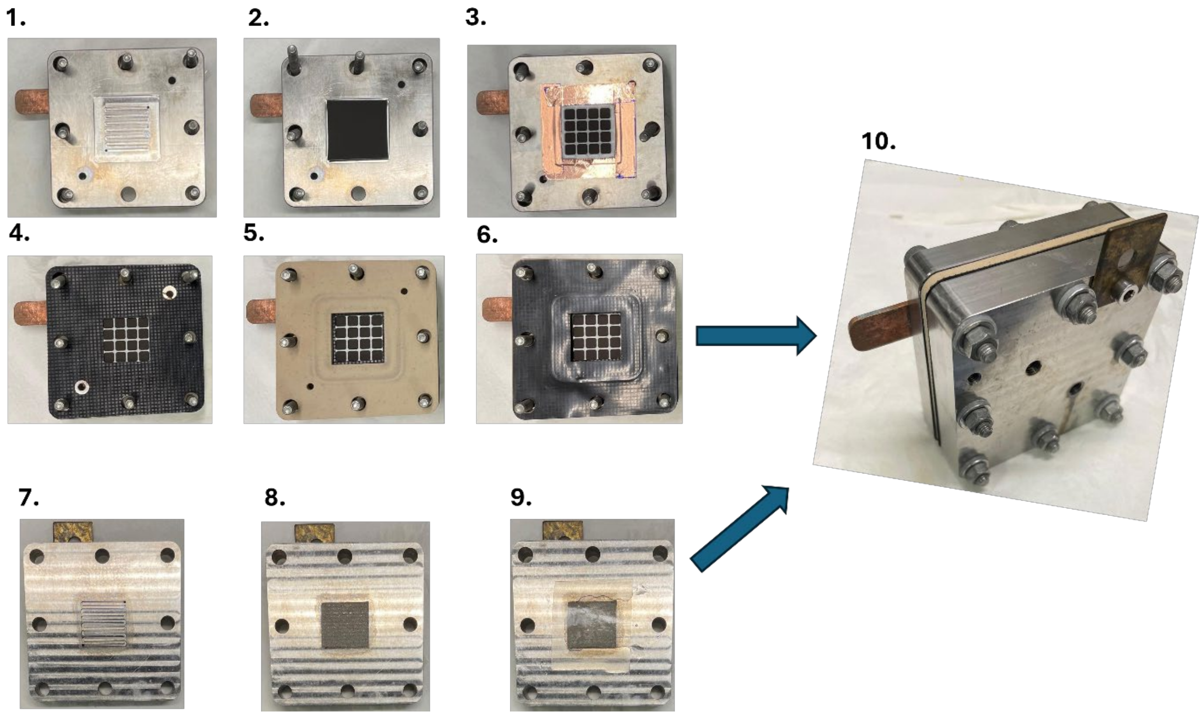

Supplementary Figure 2. Stepwise cell assembly using the grid current collector design. Steps 1.-6. show the cathode side assembly. The steps 7. – 9. the assembly of the anodic parts. The cathode side assembly (1. – 6.) is combined with the anode side assembly (7. – 9.) for the final cell assembly (10.) as shown in the image. For assembling, a torque of 3.5 Nm was applied.

a b

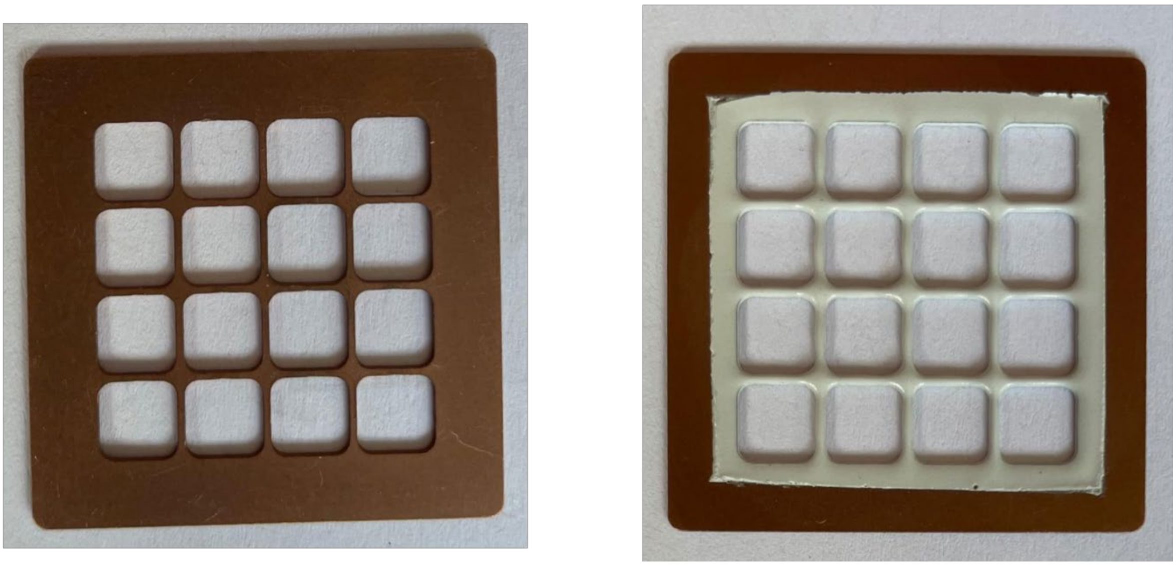

Supplementary Figure 3. Grid current collector designs. (a) The uncoated Cu grid current collector. (b) The polymer-coated current collector. The epoxide polymer was coated on all places of the grid current collector that are in contact with the liquid catholyte. The edges and backside (facing the catalyst layer) were left uncoated.

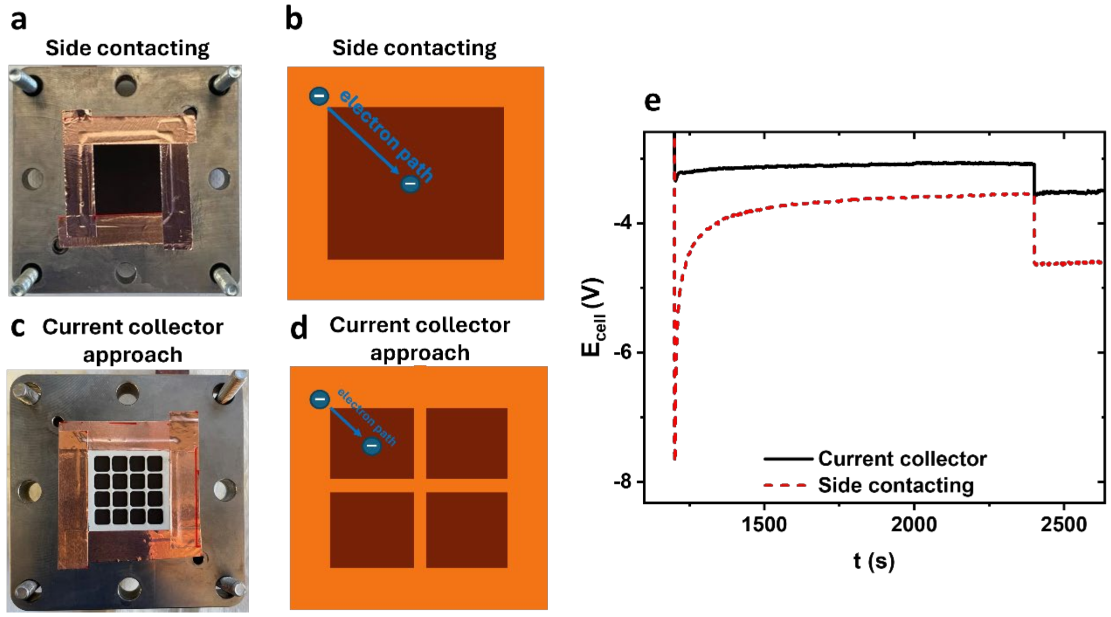

Supplementary Figure 4. Comparison of side contacting versus the polymer-coated grid current collector approach. (a) Image of a side contacted PTFE-based GDE via Cu tape. (b) Schematic of a side contacted PTFE-based GDE and showing the longest possible path of an electron from the edge of the electrode. (c) Image of PTFE-based GDE in combination with the polymer-coated grid current collector. (d) Schematic of a PTFE-based GDE in combination with a current collector. In comparison to the side contacting approach, the path of the electrons is decreased thanks to the electrode being compartmentalized into smaller segments. This enhances charge transport. (e) Cell potential response after the application of a current density of 50 mA cm^{-2} demonstrated for the side contacting approach and current collector approach. The potential response in case of the PTFE-based GDE plus current collector is much better due to a shorter electron travel pathway.

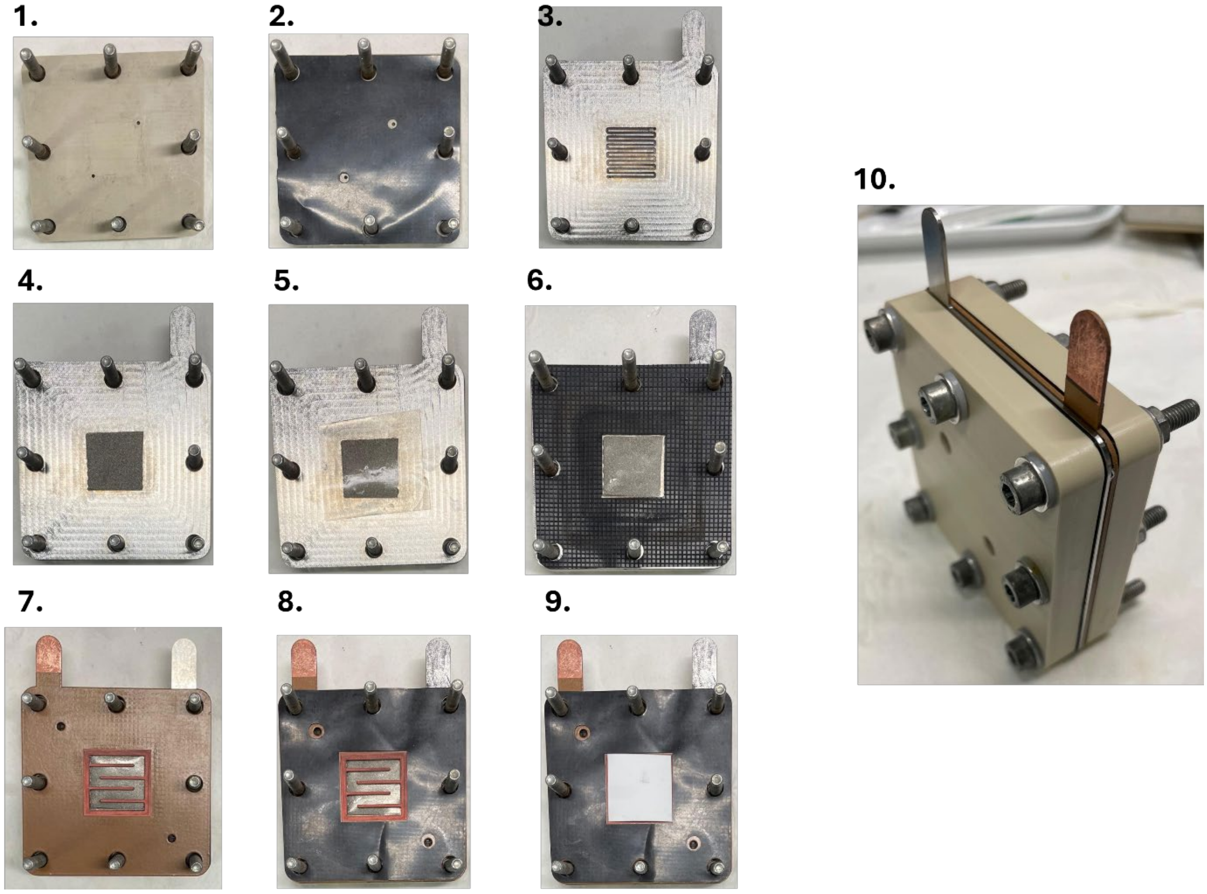

Supplementary Figure 5. Cell assembly of the monopolar plate current collector with integrated liquid flow chamber. The cell was assembled starting from the anode endplate (1.). For assembly, a torque of 3.5 Nm was applied.

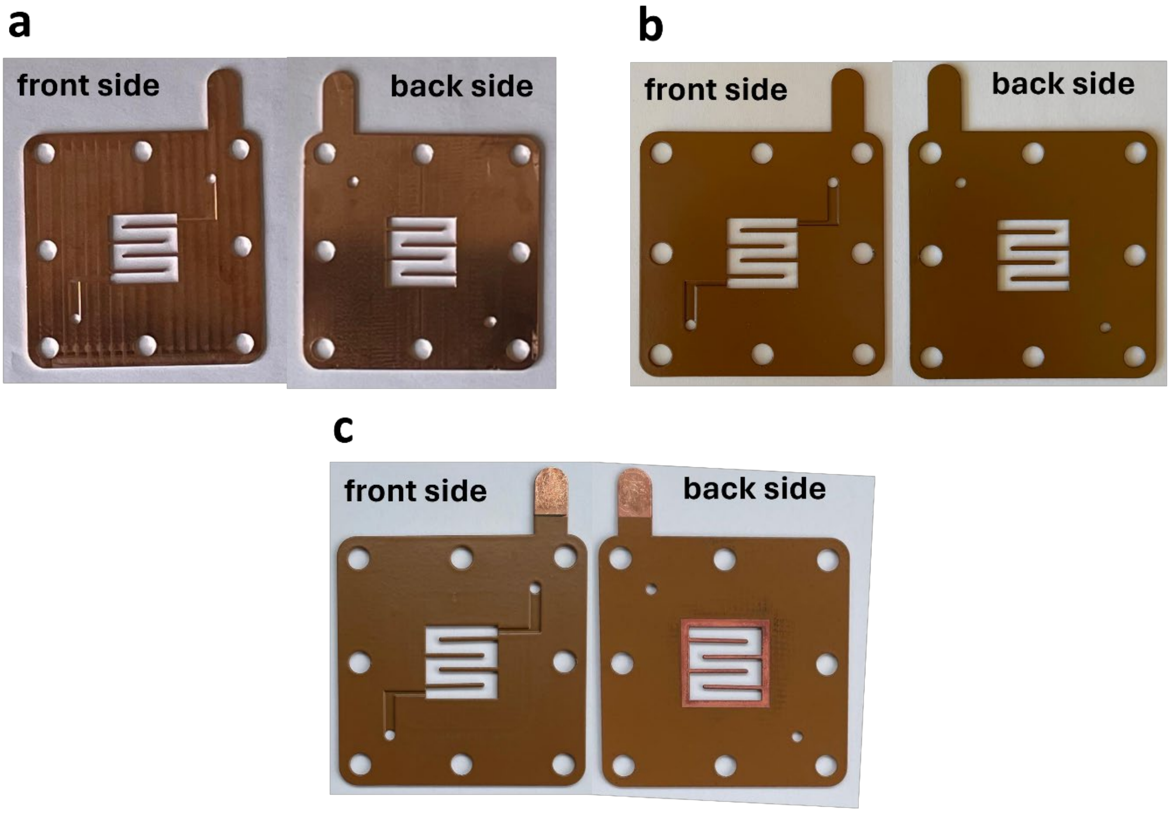

Supplementary Figure 6. Manufacturing of the monopolar plate current collector with integrated liquid flow chamber. The monopolar plate current collector is demonstrated in various stages of manufacture: (a) After CNC milling of a 1.0 mm thick Cu plate; (b) After PEEK powder coating and sintering; (c) After selective removal of PEEK coating on one side to establish homogeneous electrical connection to the catalyst layer

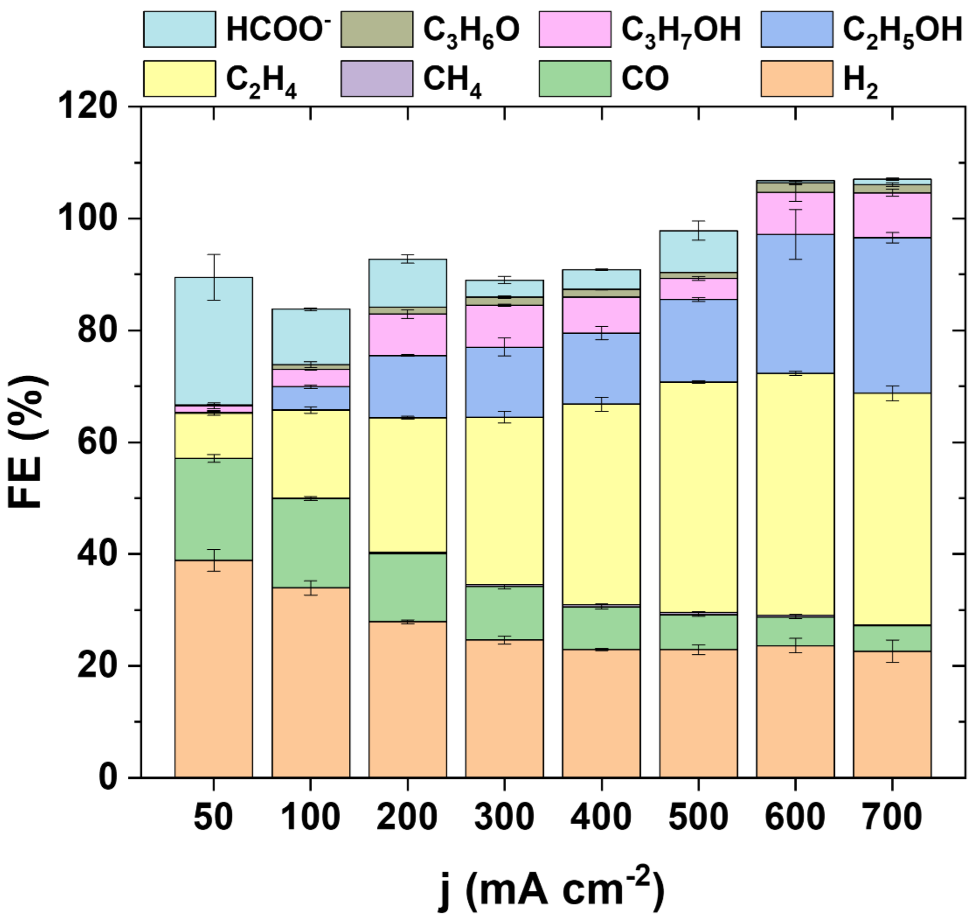

Supplementary Figure 7. Faradaic product efficiencies obtained during 5 cm^2 AEM-based CO_2 electrolysis at constant current densities employing the monopolar plate current collector design. An unsupported CuO tenorite nanoparticle catalyst (Johnson Matthey Ltd) with a loading of 1 mg cm^{-2} with 5 wt % Nafion binder was used as cathode catalyst layer. A Ni foam was used as anode. 1 M $KHCO_3$ was cycled through the cell as liquid electrolyte.

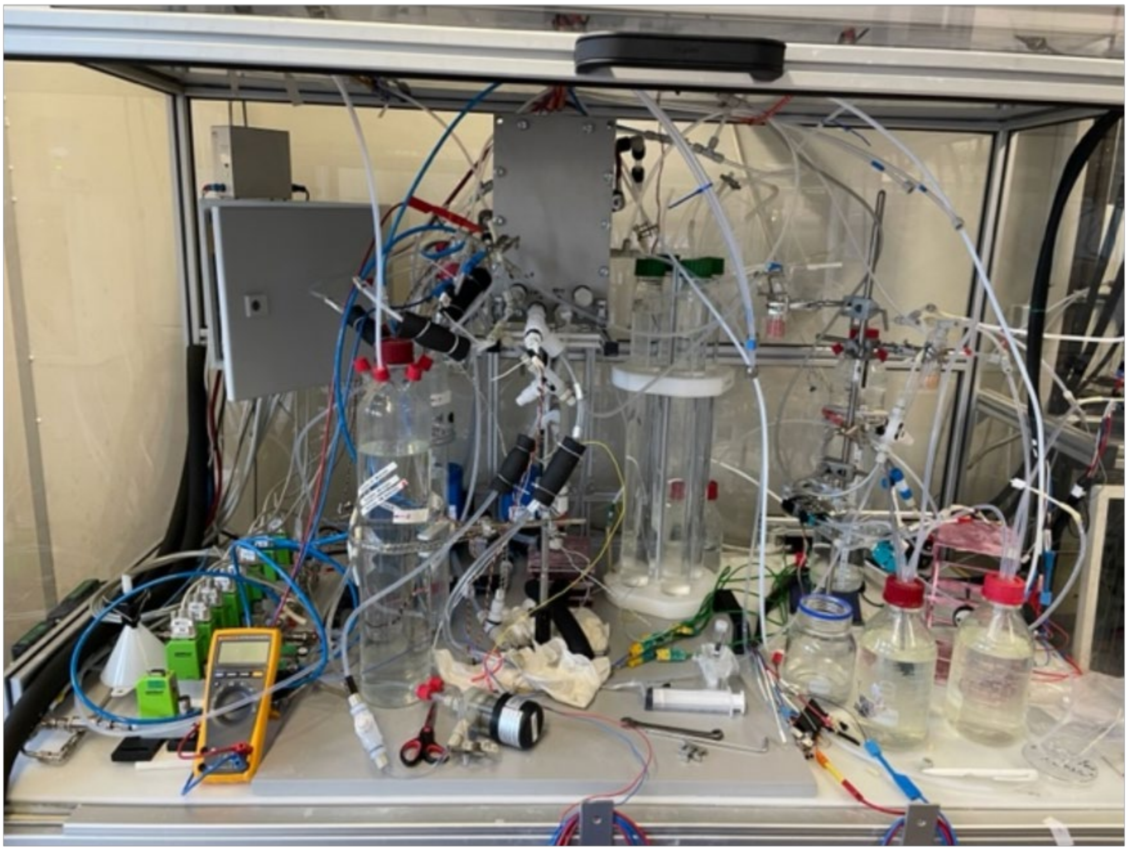

Supplementary Figure 8. CO_2 reduction Electrolyzer setup of the 100 cm^2 scale-up experiments using the new concept of a liquid flow chamber integrated monopolar plate current collector for contacting PTFE-based Gas Diffusion Electrode of 100 cm^2 geometric active surface electrode area.

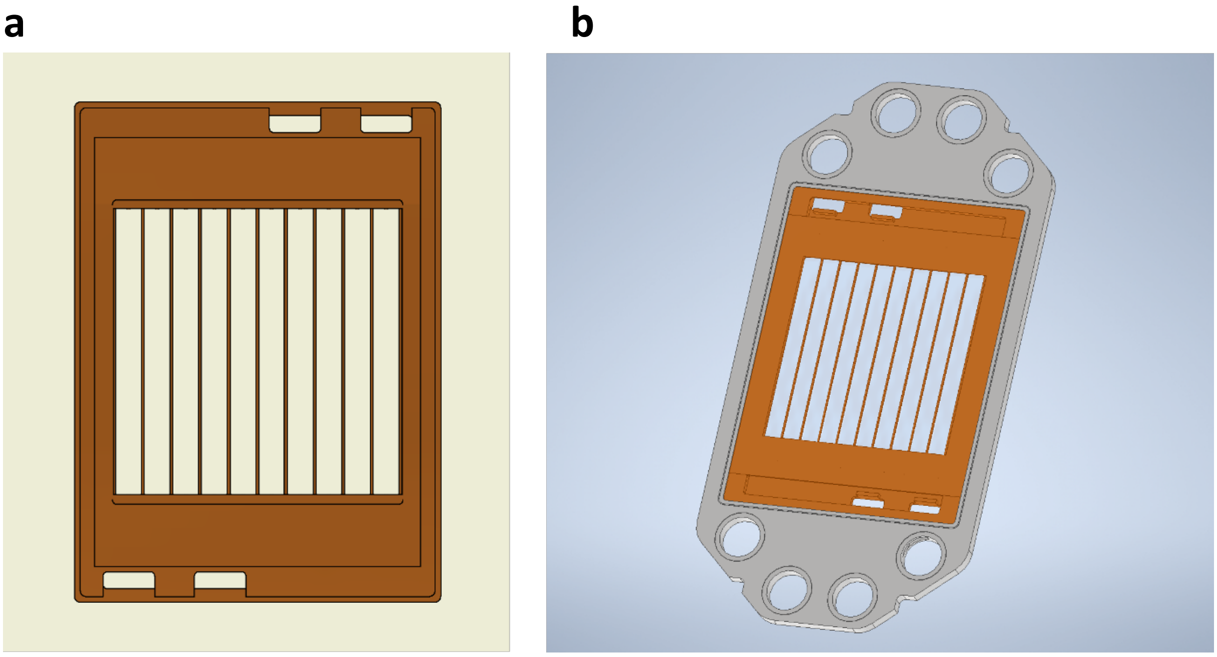

Supplementary Figure 9. **(a) 100 cm^2 parallel flow pattern and (b) corresponding framed monopolar plate current collector design for the 100 cm^2 modified commercial Electro MP Cell (https://www.electrocell.com/products/electrochemical-flow-cells/electro-mp-cell, latest access: 15.02.2024). The internally designed flow pattern and spacer with linear channels allows for supply with electrolyte at minimized flow speed and dynamic pressure loss as required for upscale. In addition, the flow pattern was made from copper coated stainless steel with PEEK coating to implement the current collector function according to the previously described 5 cm² cell versions.**

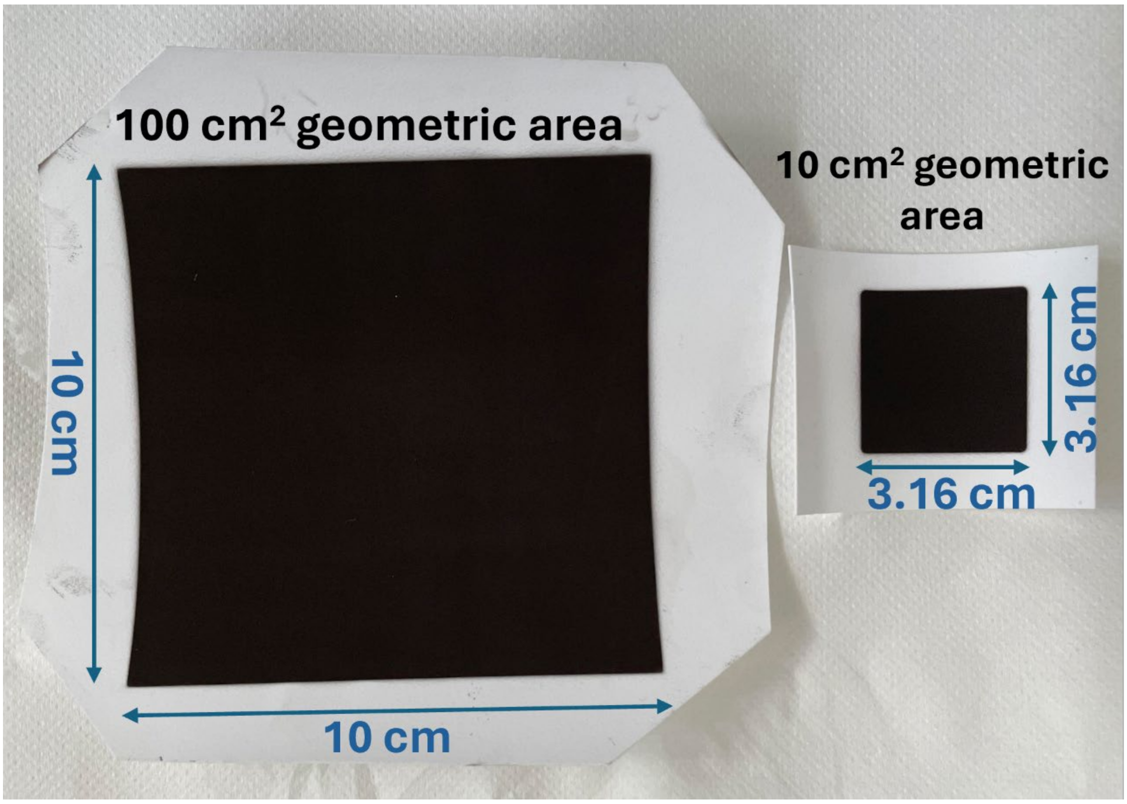

Supplementary Figure 10. As-prepared CuOx catalyst-coated PTFE porous transport layer (PTL, or Gas Diffusion layer, GDL) with 100 cm^2 (left) and 10 cm^2 (right) sprayed geometric electrode area using an air brush technique. The 10 cm^2 Porous Transport electrode (PTE, or Gas Diffusion electrode, GDE) is further cut down to 5 cm^2 prior to cell assembly.

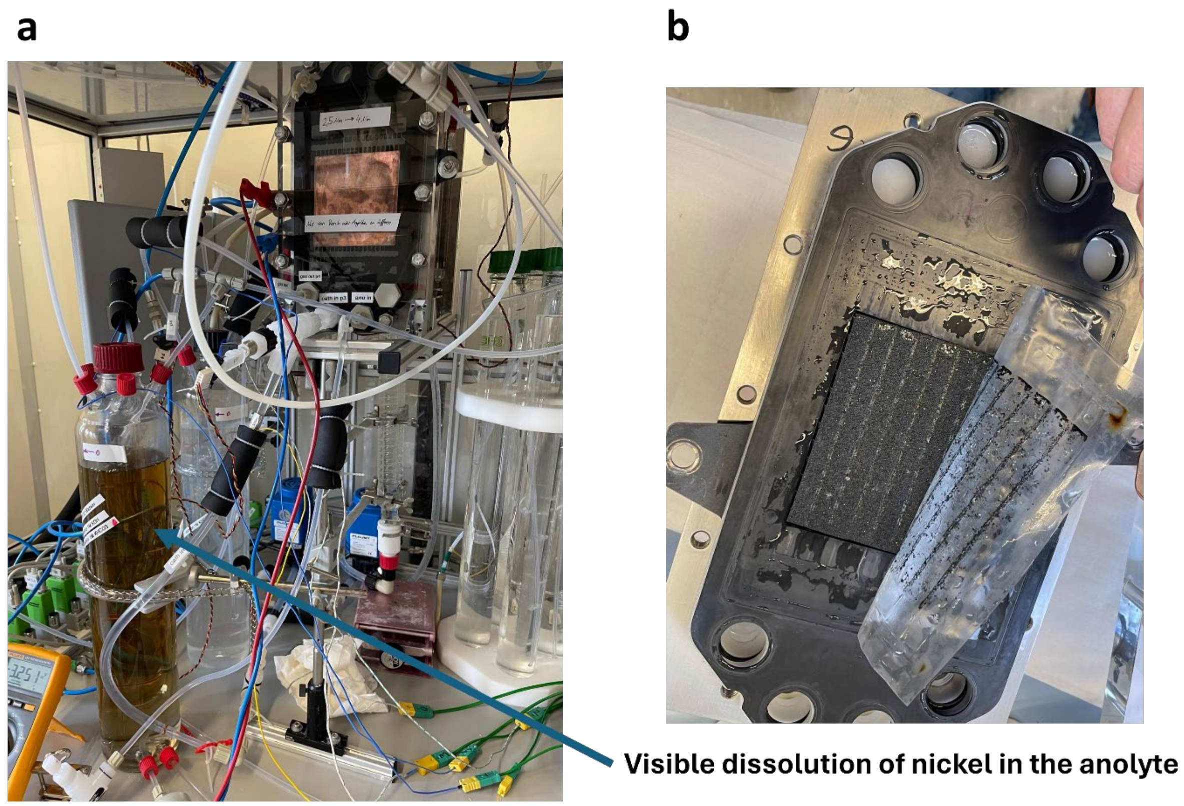

Supplementary Figure 11. Degradation of a Ni foam anode when 1 M $KHCO_3$ was used as anolyte in the AEM set up. (a) Brown-colored anolyte is visible after several minutes at constant current density, indicating material degradation. (b) Ni foam after AEM electrolyzer cell disassembly.

Supplemental Discussion 1: Stability of 5 cm^2 and 100 cm^2 PTFE GDE Electrolyzer Cells

Stability investigations of the 100 cm^2 cell equipped with the PEEK-coated current collector resulted in a stable cathode performance, while issues arose with the nickel foam anode under 1 M $KHCO_3$ feed. Ni Anode degradation occurred over time evidenced by a discoloration of the electrolyte and physical Ni foam corrosion at the end of test (Supplementary Fig. 11). This originated from the inhomogeneous anolyte flow distribution due to an open/unguided flow chamber design, leading to oxygen bubble accumulation and accelerated degradation. In addition to oxygen bubbles, CO_2 gas is transported to the anode, which further accelerates anode degradation. As mitigation, 1 M KOH was employed as anolyte which resulted in immediate stable system operation over the investigated time frame. To compare the experimental results gathered in the 100 cm^2 cell against 5 cm^2, we also used 1 M KOH as anolyte in the 5 cm^2 electrolyzer experiments.

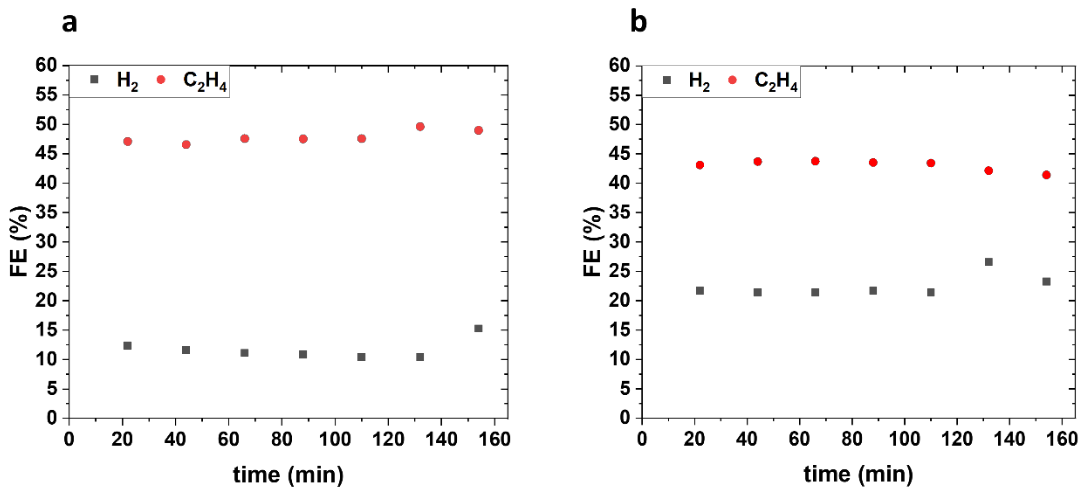

Supplementary Figure 12. Extended current hold experiments in the 100 cm^2 electrode area cell at a constant current density of 300 mA cm^{-2} using (a) the AEM setup and (b) the acidid CEM setup.
